# Study of the host specificity of PB1-F2-associated virulence

**DOI:** 10.1080/21505594.2021.1933848

**Published:** 2021-06-14

**Authors:** Joëlle Mettier, Daniel Marc, Laura Sedano, Bruno Da Costa, Christophe Chevalier, Ronan Le Goffic

**Affiliations:** a Université Paris-Saclay, INRAE, UVSQ, UMR892 VIM, Jouy-en-Josas, France; b UMR1282 Infectiologie Et Santé Publique, INRAE, Nouzilly, France

**Keywords:** Influenza virus, PB1-F2, Species barrier, zoonotic viruses, reassortment, H7N1, H3N2, mouse model, intravital imaging, chicken infection

## Abstract

Influenza A viruses cause important diseases in both human and animal. The PB1-F2 protein is a virulence factor expressed by some influenza viruses. Its deleterious action for the infected host is mostly described in mammals, while the available information is scarce in avian hosts. In this work, we compared the effects of PB1-F2 in avian and mammalian hosts by taking advantage of the zoonotic capabilities of an avian H7N1 virus. In vitro, the H7N1 virus did not behave differently when PB1-F2 was deficient while a H3N2 virus devoid of PB1-F2 was clearly less inflammatory. Likewise, when performing in vivo challenges of either chickens or embryonated eggs, with the wild-type or the PB1-F2 deficient virus, no difference could be observed in terms of mortality, host response or tropism. PB1-F2 therefore does not appear to play a major role as a virulence factor in the avian host. However, when infecting NF-κB-luciferase reporter mice with the H7N1 viruses, a massive PB1-F2-dependent inflammation was quantified, highlighting the host specificity of PB1-F2 virulence. Surprisingly, a chimeric 7:1 H3N2 virus harboring an H7N1-origin segment 2 (i.e. expressing the avian PB1-F2) induced a milder inflammatory response than its PB1-F2-deficient counterpart. This result shows that the pro-inflammatory activity of PB1-F2 is governed by complex mechanisms involving components from both the virus and its infected host. Thus, a mere exchange of segment 2 between strains is not sufficient to transmit the deleterious character of PB1-F2.

## Introduction

Influenza A viruses (IAV) are significant pathogens of humans and animals. They pose continuous health threat in the human population due to the rapid and constant evolution of seasonal strains [1]. In addition, animal strains of IAV have potential zoonotic capacities, which increase the risk of disease caused by these types of viruses. Thus, in recent years, a significant increase in human cases of H5 and H7 viruses of avian origin has been observed [2]. For example, an outbreak due to a highly pathogenic avian H7 virus observed in the Netherlands in 2003 caused conjunctivitis and influenza-like illness in 453 people [3]. Like H5N1 highly pathogenic avian influenza viruses (HPAIV), low pathogenic avian influenza viruses (LPAIV) such as H7N9 viruses can cause severe human cases and can be lethal for humans, which underlines the zoonotic capacity of LPAIV [4]. Moreover, the absence of clinical signs in infected poultry prevents the development of an efficient surveillance and allows the circulation of LPAIV strains [5]. Such sudden eruptions perfectly exemplifies the capacities of IAV to move from wild bird reservoir to humans and adapt to their new opportunistic host [2].

IAVs belong to the *Orthomyxoviridae* family, their genome is composed of eight single-stranded RNAs of negative polarity, encoding 11 to 17 proteins. Due to their segmented genome, IAVs are able to swap gene segments through reassortment [6], and the emergence of novel subtypes affecting human population generally results from one or several successive events of reassortment [7,8]. Since influenza A viruses have eight genome segments, there is theoretically a total of 254 reassortant combinations possible during cellular co-infection by 2 viruses, in addition to the two parental viruses (2^8^ = 256). However, most of these combinations are not observed due to sub-optimal compatibility between the segment-vRNA packaging signals [9]. In addition, incompatibility between viral components will exclude certain combinations (due to functional mismatch), while the fitness of the reassortant virus will also be crucial: if it is lower than that of the parental viruses, its chances to emerge are clearly reduced [10]. Interestingly, the « Asian flu » (H2N2/1957) and the « Honk Kong flu » (H3N2/1968) pandemic influenza viruses emerged via the exchange of genomic RNA segments between human and avian viruses. Both viruses received the PB1 segment from the avian parent [11], which highlights the structural and functional compatibility of this segment between strains. This segment #2 reassortment seems to have contributed to the H3N2/1968 replication and transmissibility properties [12]. Moreover, the acquisition of segment 2 from the 1918 pandemic virus by a contemporary virus has been shown to provide both a replication advantage and an increased virulence to the chimeric virus (1918 PB1: Tx/91 (1:7)) [13]. By contrast, the 2009 pandemic virus, which was a triple reassortant, had acquired its #2 segment from an H3N2 strain. Remarkably, despite its high transmissibility, this virus proved to be relatively mildly virulent [14]. Segment 2, therefore, carries virulence determinants and can be exchanged by reassortment. This segment encodes three proteins: (i) PB1, the catalytic subunit of the viral polymerase, (ii) PB1-N40 an N-terminally truncated version of this protein, and (iii) PB1-F2, a small protein with well-described apoptotic and pro-inflammatory capabilities. It, therefore, seems likely that this segment can confer a better replicative potential via PB1 and an increased virulence via PB1-F2.

Beyond highly pathogenic viruses, the genetic reassortment of avian low pathogenic viruses is also a concern. Indeed, even low pathogenic IAVs appear to be able to transmit elements that contribute to virulence by reassortment with other strains. According to the literature, the H3 subtype could be the most appropriate sub-type to support such reassortment. As a matter of fact, H3 is the most ubiquitous subtype and has a wide range of hosts, including humans, pigs, horses, dogs, cats, seals, poultry and wild waterfowl [15]. H3, together with H1 subtypes, also causes the seasonal influenza that is widely found in humans. Recently, H3N2 isolates from live poultry markets and poultry slaughterhouses in Shanghai were reported to be reassorted with H5 and H7 subtypes, within domestic ducks and wild birds [16]. The acquisition, by a human seasonal influenza virus, of segment 2 from a low pathogenicity avian virus therefore appears to be a possible event. However, the likelihood of a gain in fitness and virulence remains uncertain.

PB1-F2 is considered to be a virulence factor in mammals hosts. This protein is able to induce cell death [17] and to exacerbates the inflammation generated by viral replication [18–20]. Yet, the action of PB1-F2 on the host response is different depending on the target cell type. Indeed, its induction of cell death is mainly observed on macrophages [21,22], while the pro-inflammatory effects seem to be mainly exerted on epithelial cells [23]. However, its impact on the infected cell is subject to debate and several functions, antinomic or not, have been described. Further, it has been shown that the activities of PB1-F2 vary depending on the strain and even the host [24]. Some PB1-F2 have a more or less marked mitochondrial tropism while others are expressed in different cell compartments [25]. These differences in cellular compartmentalization could explain the opposite effects observed. Accordingly, the pro-apoptotic effects exerted by PB1-F2 on macrophages has recently been called into question [26]. Similarly, the pro-inflammatory activity of PB1-F2 is highly questioned, as it has also been described that PB1-F2 is able to inhibit the host response, especially through its inhibitory action on MAVS [27,28]. Surprisingly, these virulence functions are not observed in chickens, suggesting a specific function in the mammalian host that is absent in fowl [29; 30]. Remarkably, the deletion of PB1-F2 on a highly pathogenic H5N1 avian virus makes the virus more virulent in chickens [29]. This gain in virulence associated with a loss of expression of PB1-F2 had also been described during infection of chickens with low pathogenic H9N2 virus and is associated with a prolonged infectious shedding period [30]. All these elements show the important variability in PB1-F2 functions and underline the need to improve the characterization of this protein while distinguishing avian and human strains. In addition, the impact on host response of the introduction of an avian PB1-F2 in a human-adapted strain through a reassortment involving the segment #2 is of concern.

In this paper, we studied the functions of PB1-F2 in the context of a reassortment between a human H3N2 virus and a low pathogenic avian strain of subtype H7N1, in order to gain knowledge on the virulence capabilities of these reassortant viruses. Our data show that avian-origin segment 2 is fully functional in an H3N2 context. Furthermore, while H7N1 PB1-F2 does not cause any particular pathology in chickens, its expression in the context of infection in a mammalian model strongly exacerbates the inflammatory response. Another point identified in this work is that the transmission of PB1-F2 virulence features is not systematic and seems to be governed by other mechanisms than simple strain-to-strain transmission of viral genes.

## Methods

### Cell culture and viral infection

Chicken Lung Epithelial Cells (CLEC213) were isolated from lungs of White Leghorns chickens as described in reference [31]. Human bronchial epithelial cell-line BEAS-2B was obtained from the American Type Cell Collection (ATCC, Manassas, VA, USA). Both cell lines were cultured in Dulbecco’s Modified Eagle’s Medium: Nutrient Mixture F-12 (DMEM F/12) medium supplemented with 10% Fetal Calf Serum (FCS), 2 mM L-Glutamine and 100 UI/ml penicillin and 100 μg/ml streptomycin. Avian and human cells were maintained in a 5% CO2 incubator at 41 and 37°C respectively. For infection, cells were washed with FCS-free medium and incubated with virus at the indicated multiplicity of infection (MOI) for 2 h at 37°C or 41°C according to the cell line. Following this, the infection mixture (FCS-free medium with excess virus) was replaced by new FCS-free medium and the cells were maintained at 37 or 41°C. Besides, in the case of kinetic experiments, TPCK-treated trypsine (Worthington Biochemical Corporation, Lakewood, NJ, USA) was added to the new FCS-free medium (20 ng/ml and 0,25 µg/ml for the CLEC213 and BEAS-2B cell line respectively). Avian influenza A/Turkey/Italy/977/1999 [H7N1] and human influenza A/Scotland/20/1974 [H3N2] were used in this study. The H7N1 virus was obtained using the reverse genetic system as previously described [32,33]. H3N2 reverse genetic system was developed by viral genome extraction from BEAS-2B-infected cells by using the QIAamp viral RNA minikit (Qiagen, Venlo, Netherlands) according to the manufacturer’s protocol. Each viral segment was cloned into vector pRF483 as described previously [34,35]. Quick-change mutagenesis kit (Agilent Technologies, Santa Clara, CA, USA) was used to generate a recombinant virus containing a truncated form of Polymerase Basic protein 1-Frame 2 (PB1-F2), referred to as ΔF2. Briefly, a silent mutation in PB1 resulted in the introduction of a stop codon at the twelfth amino acid of PB1-F2 in order to abort its translation without affecting the other proteins of segment #2 [33]. Introduced mutation was confirmed by plasmid and vRNA sequencing.

### IFN-β activity measurement

Twenty-four hours before transfection, 8.10^4^ BEAS-2B cells were seeded in 24-wells plates in complete DME F/12 medium. The cells were transiently transfected using FuGENE HD (Promega, Madison, WI, USA). Transfection complex was obtained by mixing 0,5 µg DNA with 2 µL FuGENE HD in 25 µL OptiMEM medium. The cells were co-transfected with 0,3 µg of a human IFN-β luciferase-reporter plasmid [36] and 0,2 µg of a pmCherry-N1 plasmid (Takara Bio, Mountain View, CA, USA) to normalize the luciferase activity. Twenty-four hours post transfection, cells were infected (MOI = 1) and lysed at 16 hours post infection with 100 µL of passive lysis buffer (30 mM Tris pH 7.9, 10 mM MgCl2, 1% Triton X-100, 18,75% glycerol, and 1 mM dithiothreitol). Both fluorescent and luminescent reporter signals were measured in 25 µL cell lysates and using a Tecan infinite M200 PRO plate reader. For luminescence, 50 µL of lysis buffer supplemented with 1 mM ATP, 1 mM dithiothreitol and 1 µM D-luciferin (Synchem Inc, Aurora, OH, USA) were added. Each point was performed in quadruplicate and results are expressed as normalized luciferase activity relative to control.

### Quantitative RT-PCR

Total RNA was extracted from cell lysates using the RNeasy Plus kit (Qiagen) according to the manufacturer’s protocol and reverse-transcribed [25] with iScript^TM^ Reverse Transcription Supermix (Biorad, Hercules, CA, USA) using random hexamer primers. The expression levels of target genes and M1 were measured using the CFX Connect qPCR platform (BioRad) and iTaq^TM^ Universal SYBR^®^ Green Supermix (BioRad). The qPCR thermal cycle was as follows: initial denaturation 3 min at 95°C, followed by 40 cycles including 10 s at 95°C and 30 s at 60°C. Each point was performed in triplicate. To make sure that the primers produced a single and specific PCR amplification product, a dissociation curve was carried out at the end of the PCR cycle. Host genes were quantified using the comparative ΔΔCt method. The mean ΔCt obtained in mock-infected cells for each gene was used as calibrator, after normalization to endogenous HPRT, GAPDH and β-tubulin housekeeping genes. Details of the primers are provided in supplemental materials. Chicken RNA samples were processed as previously described for host response [32] and viral RNA quantifying [37].

### Ethical statements

This study was carried out in accordance with INRAE guidelines in compliance with European animal welfare regulation. The protocols were approved by the Animal Care and Use Committee at “Centre de Recherche de Jouy-en-Josas” (COMETHEA) and “Centre de Recherche de Nouzilly” (Comité d’Ethique pour l’Expérimentation Animale, Région Val de Loire) under relevant institutional authorization (“Ministère de l’éducation nationale, de l’enseignement supérieur et de la recherche”). Authorization number: mice experiments 2015100910396112v1 (APAFIS#1487), chickens’ experiments: approval number 2010/7. All experimental procedures were performed in a biosafety level 2 (mice) or biosafety level 3 (chickens) facilities.

### Animals and eggs

Female BALB/c mice were purchased from the “Centre d’Elevage R. Janvier” (Le Genest Saint-Isle, France) and were used at 8 weeks of age. NF-κB-Luciferase transgenic Balb/C mice were obtained by backcrossing NF-κB-Luciferase Transgenic B10.A (kind gift of Pr Richard Flavell, Howard Hughes Medical Institute) with Balb/c mice to ensure the production of transgenic mice with white fur and hence avoid the light absorption due to the dark one of B10 mice. Mice were housed in negative pressure isolators in a containment level 2 facility. Food and water were available ad libitum. Mice were lightly anesthetized with a mixture of ketamine and xylazine (60 mg/kg and 12 mg/kg, respectively) and inoculated intranasally with the indicated dose of virus in 50 µL PBS. The conditions of the mice were monitored daily. Humane endpoint was used during survival study: mice were euthanized via cervical dislocation when body weights were reduced to 75% of the starting weights or when the rectal temperature was inferior to 32°C. Four-week-old specific-pathogen-free (SPF) histocompatible B13/B13 White Leghorn chickens were housed in biosafety level 3 cabinets under negative pressure with HEPA-filtered air. Briefly, three groups of 23 (WT H7N1), 26 (ΔF2 H7N1) and 10 (mock) birds were inoculated intra-tracheally with 5 × 10^5^ EID50 (0.1 ml) of each virus, along with 10^6^ EID50 (0.2 ml) in the choanal cleft, while in the mock group the virus suspension was replaced by PBS. Birds were carefully monitored daily during the course of the trial (one week). Four birds at days 1, 2, 3 and 4 post-inoculation (p.i.) and 1–3 birds at day 7 p.i. for each virus-inoculated group were euthanized and necropsied, along with two mock-inoculated birds at each of the indicated times. Swabs and tissue samples from lung, kidney, and brain were collected from each animal and frozen at −80°C until use for further downstream analyses (viral and host RNA quantification). SPF fertilized chicken eggs (PA12 White Leghorn strain) were purchased from the “Plate-forme d’Infectiologie Expérimentale – Centre INRAE Val de Loire” (Nouzilly, France). Upon arrival, the eggs were placed in an incubator and then infected at day 11 of embryonic development with 50 PFU of WT or ΔF2 H7N1 Nluc or mock-infected. Viruses were diluted in PBS containing 100 UI/ml penicillin and 100 μg/ml streptomycin. Inocula were injected into the allantoic cavity (0,1 ml per egg). Eggs were processed for IVIS imaging at 24 h and 48 hours pi.

### IVIS monitoring

Bioluminescence quantifications of the firefly luciferase (NF-κB-Luciferase transgenic mice) and the nanoluciferase (recombinant viruses) were achieved using the IVIS 200 imaging system (Perkin Elmer). Mice were anaesthetized and luminescence was measured 1 min after intra-nasal injection of 50 μl of PBS containing D-luciferin (7 mg.kg^−1^, Synchem) or 50 µl of PBS containing furimazine (1:50 dilution of the Promega NanoGlo Luciferase Assay Substrate). For chicken embryos imaging, 300 µl of furimazine (1:20 dilution in PBS of Promega substrate) was injected into allantoic cavity of living eggs. Five minutes post-injection, the embryos were extracted and washed several times in PBS to remove allantoic fluid. Embryos were then imaged using the IVIS device. Living Image software (version 4.0, PerkinElmer) was used to measure the luciferase activities. A digital false-color photon emission image of the mouse was generated, and photons were counted within the whole-body area (firefly), airway area (nanoluc) or the embryo surface (eggs). Photon emission was measured as radiance in p.s^−1^.cm^−2^. Sr^−1^.

## Results

### In vitro *virus characterization*

To facilitate the analysis of the replication of the H7N1 and H3N2 viruses under study, we inserted a nanoluciferase (Nluc) cassette into the segment 2 of these viruses. The two Nluc-tagged viruses were designed using the strategy described by Tran and collaborators [38]. Sequences of the two engineered segments are available in supplemental material. We have also produced mutant viruses unable to express PB1-F2 (ΔF2) in each strain, through introduction of a stop codon at position 12 of segment #2-frame #2. These mutations do not alter the coding sequence of PB1.

**Figure 1.**
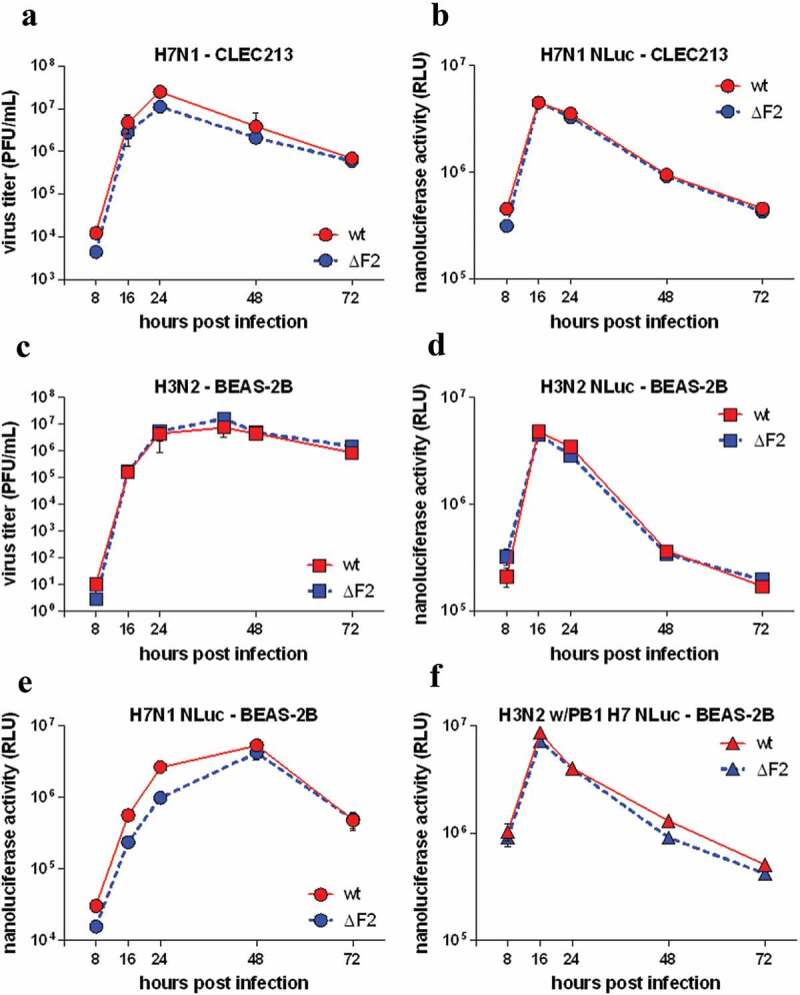
In vitro replication kinetics comparisons. Monolayers of CLEC213 and BEAS-2B cells were infected for 72 hours at MOI 0.01 with conventional H7N1 (A), NLuc H7N1 (B), conventional H3N2 (c), NLuc H3N2 (D). BEAS-2B cells were infected with NLuc H7N1 or the reassortant NLuc H3N2 virus expressing PB1 segment from H7N1 (E and F). PFU production (A and C) and NLuc activity in cell lysates (B, D, E and F) were quantified over time. Results represent the mean ± SEM of triplicate wells of a representative experiment

As shown in Figure 1, WT and ΔF2 viruses replicate similarly in both avian and mammal hosts. In the avian CLEC213 cells, the replication of the ΔF2 H7N1 virus perfectly mirrors that of the parental WT virus, reaching the peak of virus production in the supernatant at 24 hours (Figure 1A), while the maximum of luminescence emitted by the infected cells was observed at 16 hours (Figure 1B). In the H3N2 virus-infected human BEAS-2B cells, the virus titer in the supernatant reached a plateau at 24 hours (Figure 1C) while the luminescence emitted by the infected cells also peaked at 16 hours (Figure 1D). The decrease of the viral titer after 24 hours in the supernatant of the CLEC213 cells (Figure 1A) probably reflects a lower stability of the H7N1 viral particles in the warm cell culture medium (41°C), as compared to that of the H3N2 virus in the supernatant of the BEAS-2B cells (Figure 1C). Thus, the deletion of PB1-F2, whether in the avian H7N1 or in the human H3N2 virus, does not impair the virus growth in the cognate system of cultured cells. Interestingly, the peak of luminescence, which precedes by about eight hours the peak or the plateau of the viral titer in the supernatant, probably reflects the maximum activity of virus replication inside the infected cells. Thereby, we chose to rely on the kinetics of NLuc luminescence to monitor the viral replication in next experiments. We first compared the replication of the WT and ΔF2 H7N1 avian viruses in human BEAS-2B cells. As shown in Figure 1E, the peak of luminescence is considerably delayed as compared to that observed in avian cells with the same viruses (Figures 1B, 48 hours vs. 16 hours), probably reflecting the sub-optimality of the human cell context for the avian virus. It should be noted that in this configuration, the deletion of PB1-F2 in the avian virus causes a slight decrease, both of the luminescence signal before the peak (Figure 1E) and of the virus titer in the supernatant (not shown). Finally, in order to examine the outcome of a plausible viral reassortment, we assessed the compatibility of avian virus segment #2 in the background of a mammalian virus by producing a pair of chimeric viruses by reverse genetics: Nluc H3N2 w/PB1 H7 and Nluc H3N2 w/PB1ΔF2 H7. The profile of the viral growth curve, as monitored by the NLuc activity (Figure 1F), is very close to that of the H3N2 virus (Figure 1D), and here again, the deletion of PB1-F2 has no impact on viral replication. The H7N1 virus is therefore capable of replicating abundantly within mammalian cells and its segment #2 is compatible with the backbone of a human H3N2 virus. The absence of PB1-F2 expression has little or no effect on in vitro replication.

### Host response analysis in vitro

**Figure 2.**
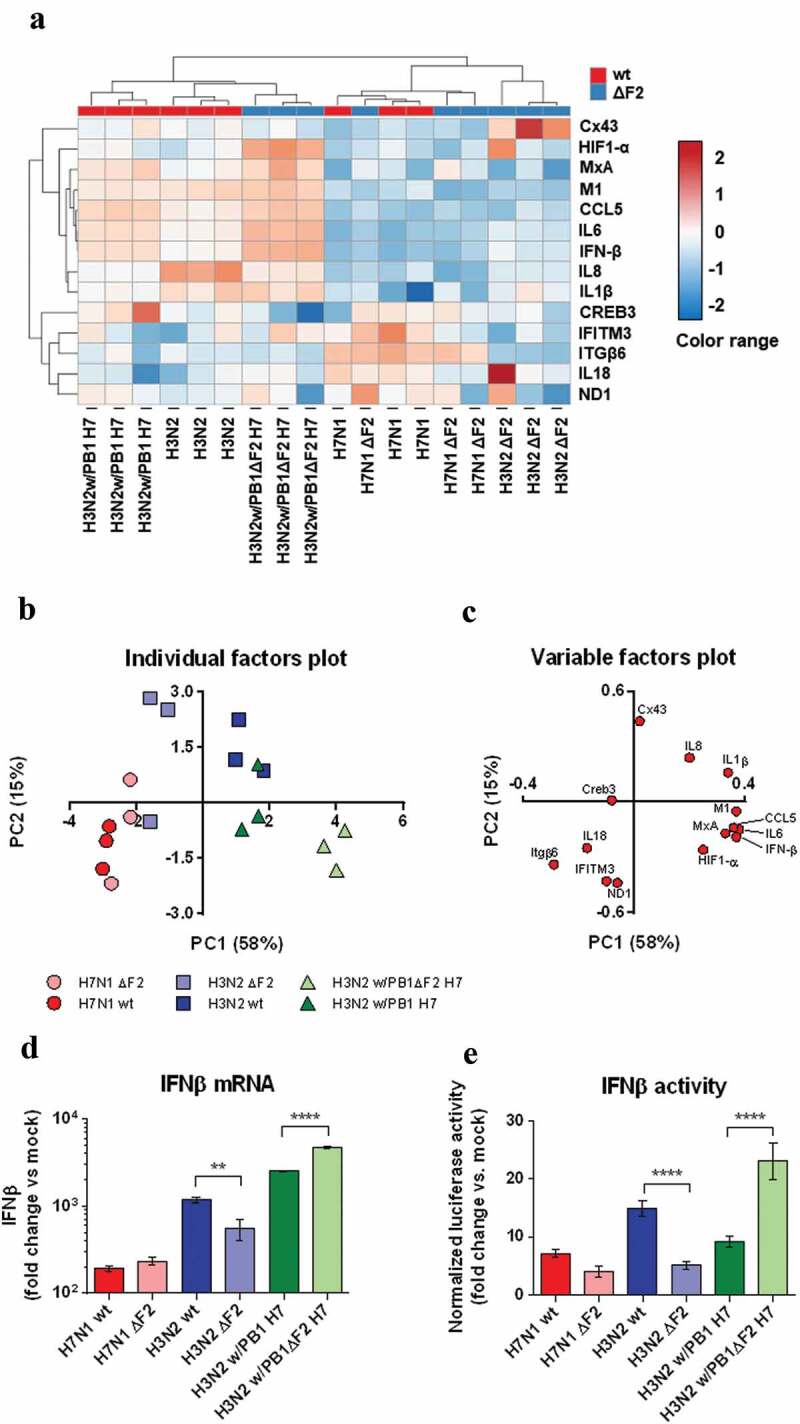
BEAS-2B host response characterization. BEAS-2B cells were infected with H7N1, H3N2, reassortant virus H3N2w/PB1 H7 and their respective PB1-F2 deficient mutants (ΔF2) for 8h at MOI 3. After RNA samples processing, qRT-PCR were performed to quantify expression of 14 transcript: MxA, IFN-β, IFITM3, CCL5, IL8, IL6, IL1β, IL18, Cx43, ITGβ6, HIF1-α, Creb3, ND1 and M1. Heat map was created by using the ClustVis tool [57]. Both rows and columns are clustered using correlation distance and average linkage. We used mRNA quantifications (as fold change vs control) to plot the heat map by using ClustVis tool. Coefficients were applied to fold changes so that values are centered and range from −2 to +2 as extreme values.(A). Principal component analysis was performed using ClustVist, individual factors plot (B) and variable factor plot (C) are represented. IFN-β mRNA relative quantification by qRT-PCR. (D). BEAS-2B cells were transfected with IFN-β -luciferase reporter and then infected for 16 h at MOI 1 with the different viruses (E). Histograms are the means ± SEM of 3 (qRT-PCR) or 4 independent samples (IFN-β activity). Data represented are representative of at least three independent experiments. **: p-value = 0.0055; ****: p-value < 0.0001 (ANOVA)

We then aimed to characterize the effects of H7N1- and H3N2-origin PB1-F2s on host response. We infected BEAS-2B cells with the three virus pairs described above. We used high MOI in this case to synchronize the infection of the cell layer. We evaluated the response of the infected cells, as assessed by the qRT-PCR-measured expression of 13 cellular genes, along with the quantification of the viral M gene. Cellular genes were selected on the basis of their antiviral (MxA, IFN-β, IFITM3), inflammatory (CCL5, IL8, IL6, IL1β), immune (IL18) functions, or for their role in cell integrity (Cx43, ITGβ6, HIF1-α, Creb3). Mitochondrial gene ND1 was also included because of its involvement in the H7N1 infectious process [33]. Surprisingly, this expression analysis, which was carried out in parallel for all six viruses, revealed that the viruses were grouped into two distinct clusters: the H3N2 virus and the two chimeric viruses, on the one hand, and the two H7N1 viruses and the H3N2 ΔF2 virus, on the other hand (Figure 2A and B). We performed principal component analysis (PCA) in order to highlight the differences between the studied viruses according to functional gene groups (Figure 2B and C). In Figure 2B, each sample has been plotted in a two-dimensional system whose axes are defined as a linear combination of our originals variables. The variable factors plot in Figure 2C show which variables contribute to define the axes. The dimensions determined by the PCA explain 73% of the intergroup variability (58% explained by the first dimension PC1 and 15% by the second one PC2). The dimension 1 is defined by both inflammatory and antiviral factors (Figure 2C). Thus, samples positioned to the rightmost side of the x-axis in Figure 2B (i.e. WT H3N2 and the two chimeric viruses) indicated a strongest inflammatory and antiviral response. It can therefore be seen that the two H7N1 viruses are grouped together, indicating that their PB1-F2 is neither involved in the inflammatory nor antiviral response in BEAS-2B cells. On the contrary, the two H3N2 viruses are dissociated by the abscissa axis, the virus expressing PB1-F2 being moderately more inflammatory. Finally, for the chimeric virus, the deletion of PB1-F2 exacerbates the inflammatory response, which seems at first instance counter-intuitive given the results obtained for the other two viruses. The function of PB1-F2 is therefore highly dependent on the host/virus environment in which it is expressed.

The qRT-PCR data obtained with IFN-β gene are detailed in Figure 2D. In order to validate this gene expression information in functional terms, we used a reporter plasmid of type 1 IFN activity transfected into BEAS-2B cells (Figure 2E). Functional IFN activity was measured after 16 h pi (instead of 8h pi for mRNAs) to allow accumulation of the reporter gene within the infected cells. We observed that the IFN activity correlated with the level of IFN-β gene expression: the activity is reduced for the ΔF2 H3N2 virus compared to the WT virus while it is increased in the case of infection by the chimeric virus lacking PB1-F2 compared to the chimeric WT virus. Of note, as replication kinetics are slightly different between H7N1 and H3N2 viruses in BEAS-2B cells, comparisons between H7N1 and H3N2 viruses can be hazardous, so only comparisons between WT and DF2 viruses should be considered here. In conclusion, the H7N1 virus, despite its replication ability in BEAS-2B cells, is relatively non-inflammatory and the expression of PB1-F2 does not influence the inflammatory response unlike what is observed with the H3N2 virus. In addition, and unexpectedly, when cells were infected with the H3N2 w/PB1 H7 virus, PB1-F2 expression reduced the inflammatory response.

### PB1-F2 expression does not modulate H7N1 virulence during chicken infection

**Figure 3.**
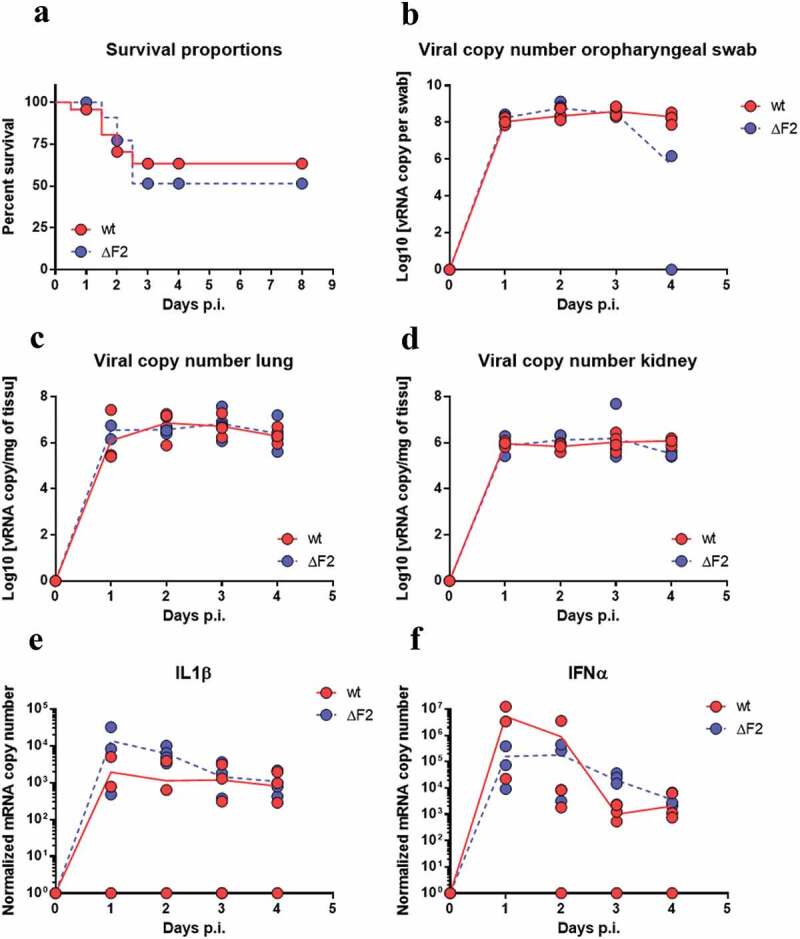
Time-course of dynamic parameters in chickens infected by WT- or ΔF2-H7N1. (A) Chickens survival curves (wt n = 23; ΔF2 n = 26). (B, C and D) qRT-PCR viral quantification in oropharyngeal swabs, lungs and kidneys. (E and F) qRT-PCR quantification of IL1-β and IFN-α inductions in lungs of infected chickens. Curves represented are the mean values obtained from four animals

**Figure 4.**
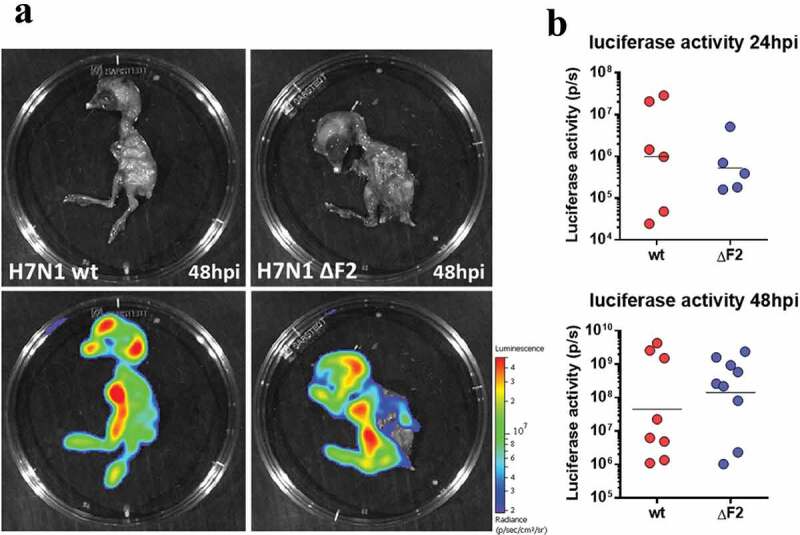
Infection of embryonated eggs with WT and ΔF2 Nluc H7N1 viruses. Fertilized chicken eggs were infected at day 11 of embryonic development with 50 PFU of WT or ΔF2 Nluc H7N1 viruses. Eggs were processed for IVIS imaging at 24 and 48 h pi. Bioluminescence was acquired 5 min after injection of furimazine in the allantoic cavity (A) Embryos picture (top panel) and overlay of the NLuc signal (bottom panel) at 48 h pi. The scale on the right indicates the average radiance (sum of the photons per second from each pixel inside the ROI/number of pixels) p/sec/cm2/sr, number of photons per second per square centimeter per steradian. (B) Quantification of the NLuc signals at 24 and 48 h pi. Data are represented as the total flux in each pixel (p/s) integrated over the whole surface of the embryos. Between 5 and 10 eggs were monitored for each condition. Bars represent the geometric mean of a representative experiment performed twice

We next assessed the contribution of PB1-F2 to the viral pathogenicity in the more realistic situation of an experimental infection of chickens with the avian H7N1 virus. 25-day-old specific-pathogen-free (SPF) White Leghorn chickens were inoculated with 5 × 10^5^ EID_50_ through intra-tracheal route combined with 10^6^ EID_50_ through oral route (choanal cleft). 23 chickens were infected with the WT H7N1, 26 chickens with the ΔF2 H7N1 and 10 chickens were mock-infected. As shown in Figures 3A, 40 to 50% of the virus-infected chickens died between 24- and 72-h post-infection, with no obvious effect of PB1-F2 deletion on the survival curve when compared to the WT virus. This type of result has already been described when inoculating chickens with a low dose of low pathogenic H9N2 virus [30]. In both oropharyngeal swab (Figure 3B) and lungs (Figure 3C), the maximum viral load was observed on days 2 and 3 pi, as previously observed in the same model [32,37]. We notice that this peak of viral load coincides with the peak of disease and death in the chickens. A high viral load was observed in the kidney (Figure 3D), reflecting virus transport by the blood. Overall, we observed no noticeable difference between the two viruses as regards the viral loads (Figure 3B–D). Finally, the intensity of the host response was studied by quantifying the expression of a pro-inflammatory mediator (IL1β), and an anti-viral mediator (IFN-α). Here again, few biologically relevant differences are noteworthy (Figure 3E and F), suggesting that the function of PB1-F2 is neither involved in viral replication nor in modulation of the host response in chickens. Due to biosafety considerations, we were unable to perform intravital imaging on chickens. We therefore tested the replication of WT and ΔF2 H7N1 viruses in embryonated eggs. The different experiments conducted did not allow us to identify differential tropism between viruses expressing or not PB1-F2. As shown in Figure 4A, viral replication is observed throughout the whole embryo with particularly intense signals in the brain and gut. However, the same replication pattern is observed for both viruses. Moreover, we found no difference in the egg-based replication of the 2 viruses, whether as assessed by the luciferase activity (Figure 4B) or by the hemagglutinin activity measured in the allantoic fluids that were collected at four days p.i. (not shown). In addition to the measurements made on embryos, analysis of luciferase signals from whole eggs as well as allantoic fluids also did not identify any differences in replication between the two viruses (data not shown).

Altogether, our avian in vivo data, both in the chicken model and in the embryonated egg, indicate that PB1-F2 from a H7N1 LPAIV has no impact on virus replication, biodispersion and shedding, and does not alter host response.

### Replication of H7N1 in mice

**Figure 5.**
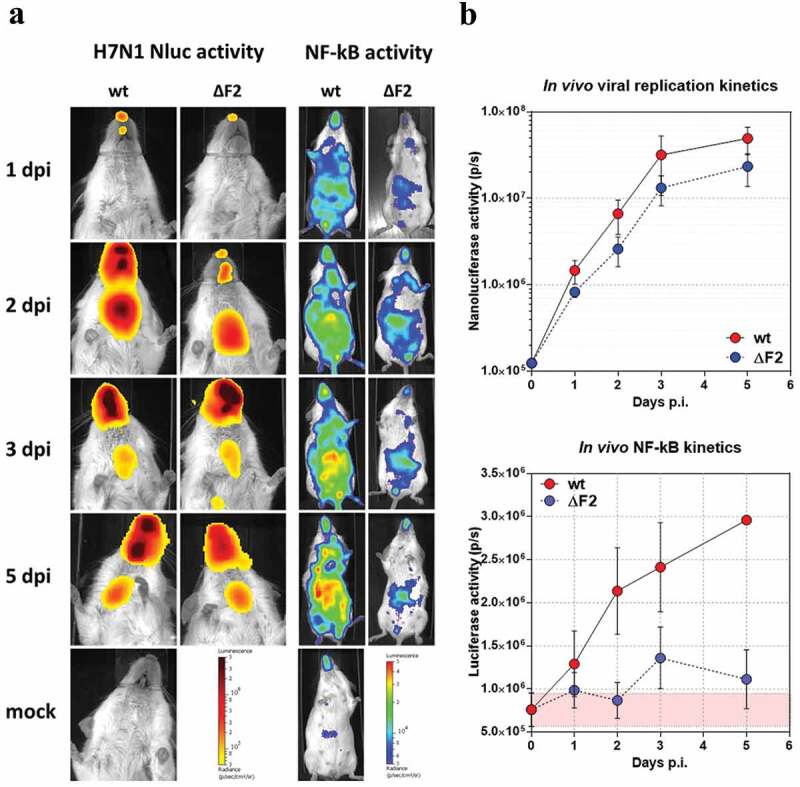
Kinetics of viral replication and induced inflammation in NF-κB-luciferase mice infected with WT or ΔF2 NLuc H7N1 viruses. NF-kB luciferase mice were infected with 10^5^ PFU of WT or ΔF2 Nluc H7N1 intranasally. (A) Bioluminescence was measured by intranasal inoculation of furimazine or D-luciferin and using an IVIS system. NLuc signals (viral replication) were only observed in the nose and chest (left panels). Firefly luciferase (NF-κB activity) was monitored in the whole body (right panels). The color scales indicate the average radiance (sum of the photons per second from each pixel inside the ROI/number of pixels) p/sec/cm2/sr, number of photons per second per square centimeter per steradian. (B) Time course of NLuc activity is represented in the upper panel, time course of firefly activity is shown in the lower panel. Bioluminescence activities were quantified using Living Image software. Data are represented as the total flux in each pixel (p/s) integrated over the whole surface of the respiratory tract (NLuc) or the mouse (firefly). Curves are the mean ± SEM values obtained from five animals and are representative of at least three independent experiments

H7N1 viruses are not widely described for their zoonotic capabilities. However, evidence of anti-H7 antibodies has been observed in serum samples collected from poultry workers. In addition human cases of infection with H7N9 viruses indicate that H7 viruses can, although rarely, infect humans [39,40]. Moreover, H7N1 subtypes isolated from ostrich and chicken were able to induce mortality and morbidity in mouse and ferret models without any prior adaptation, supporting a minimal level of zoonotic potential of H7N1 viruses [41–44]. To study further the effect of PB1-F2 in a potential species barrier crossing, we infected mice with various doses of our WT NLuc H7N1 strain. The lethal dose 50% was estimated to be 7 × 10^3^ PFU per mouse. Then, we used transgenic NF-κB-luciferase mice to study the host response through inflammatory induction [18]. The WT H7N1 and the ΔF2 H7N1 displayed comparable tropisms and kinetics of replication in mice airways (Figure 5A–left panels). However, it should be mentioned that the ΔF2 virus replicated to slightly lower levels than the WT virus (1.7 to 2.5 times less, although not statistically significant; Figure 5B upper panel). The inflammatory signal was also monitored during the infection with both viruses. Yet, this signal does not evolve in the same way as that of replication since the inflammatory process is clearly more pronounced in mice infected with the WT virus than in mice infected with ΔF2 (Figure 5A–right panels). Indeed, mice infected with the PB1-F2-expressing virus show a continuous increase in inflammatory activity over time (Figure 5B–lower panel) while mice infected with ΔF2 developed only moderate inflammation. The avian PB1-F2 of the H7N1 virus therefore contributed to induce or to increase the inflammation developed during viral infection of a mammalian organism, even though it had no effect in vivo in chicken nor in an in vitro model of mammalian epithelial cells. These data highlight the host specificity of PB1-F2 as well as its need to be expressed within a complex tissue rather than in simple cell culture to reveal its virulence functions.

### Pathogenesis of a chimeric H3N2 harboring an avian segment #2

**Figure 6.**
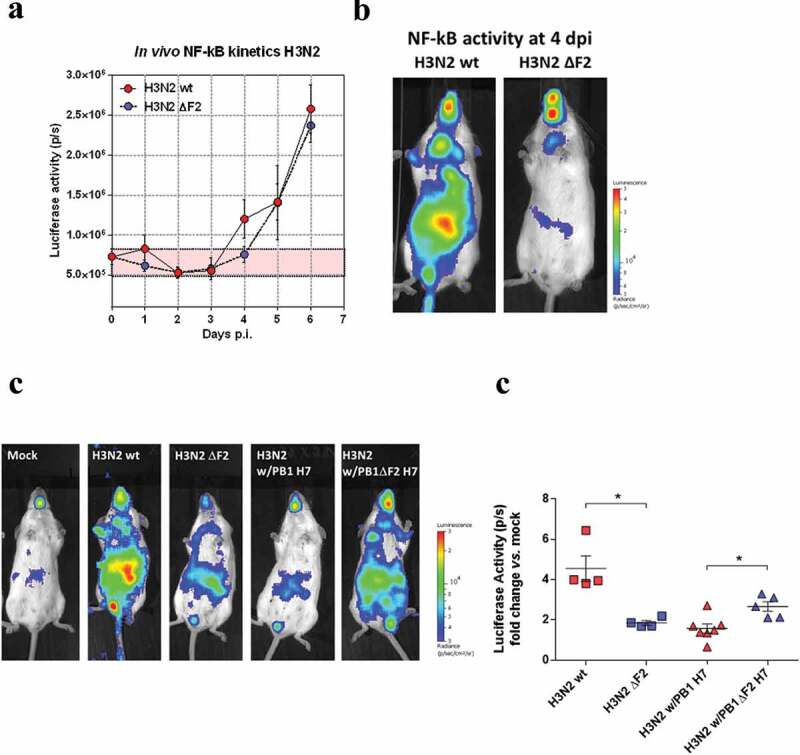
NF-κB activity of mice infected with H3N2-NLuc or reassortant. Bioluminescence was measured by intranasal inoculation of D-luciferin and using an IVIS system. Scales on representative pictures indicate the average radiance (the sum of the photons per second from each pixel inside the ROI/number of pixels) p/sec/cm2/sr, number of photons per second per square centimeter per steradian. Bioluminescence activities were quantified using Living Image software. Data are represented as the total flux in each pixel (p/s) integrated over the whole surface of the mouse. (A) Time course of the inflammation induced after infection of NF-kB-luciferase mice with 30 PFU of H3N2 WT or ΔF2 (n = 6). (B) Representative pictures of H3N2 WT or ΔF2-infected NF-kB-luciferase mice at day 4 pi. (C) Representative pictures of the inflammation induced after infection of NF-κB-luciferase mice with 30 PFU of H3N2 or H3N2 w/PB1 H7 and their ΔF2 counterparts (n = 5). (D) NF-kB-luciferase activity quantification of infected NF-kB-luciferase mice. *: p-value < 5% (Mann–Whitney test)

To further investigate our data obtained on PB1-F2 with the H7N1 virus, we evaluated the effect of H3N2 viruses (WT vs. ΔF2) on NF-κB Luc mice. Based on the measured NF-κB Luc activity, the kinetics of inflammation was different from that observed in H7N1-infected mice, being apparent only from the 4th day pi and rapidly becoming very intense (Figure 6A). At day 6 pi, the inflammatory levels reach those observed for the H7N1 virus. Contrary to what we observed with the H7N1 virus, the inflammation kinetics of the two viruses were very similar, except for a slight augmentation observed at day 4 pi with the WT virus (Figure 6A and B). This type of effect associated with PB1-F2 has already been observed for other viral strains, such as H1N1 [20]. We then chose to focus on this time point to compare the effects of PB1-F2 in H3N2 viruses and H3N2 w/PB1 H7 chimeric virus. We confirmed the marked differences between the WT and ΔF2 H3N2 viruses (Figure 6C) since the level of inflammation was two times more pronounced in WT-infected mice (Figure 6D). Surprisingly, while it allows the expression of the avian PB1-F2 with a strong pro-inflammatory capacity, the chimeric virus expressing the H7N1 segment #2 was less inflammatory than the WT H3N2 virus. Importantly, there is no bias related to viral replication since all viruses used in this experiment replicated to equivalent levels (sup Figure 1). Finally, the chimeric virus devoid of PB1-F2 induced a higher inflammatory response than the non-mutated chimeric virus. Though unexpected and counter-intuitive given our previous results, this increased inflammatory response corroborates the data obtained in vitro, especially with regard to the IFN activity (Figure 2D and E).

These results suggest that the pro-inflammatory responses induced by PB1-F2 are governed by complex mechanisms in which other viral proteins or strain-specific mechanisms are clearly involved. Thus, and contrary to our initial hypothesis, the transmission of the inflammatory and host deleterious character of PB1-F2 proves to be much more complex than a mere segment exchange.

## Discussion

In this work, we used a low pathogenicity avian influenza virus of H7N1 subtype. This virus is considered as one of the precursors to the HPAI strains that caused a major epizootic in Italy in the 2000s [32,45]. It appears that this virus is naturally well adapted to replication in mice and ferrets, since without prior adaptation, it can kill animals at relatively low doses. Indeed, mice are not naturally infected with influenza viruses and most strains require prior experimental adaptation to mice through successive passages from lung to lung [46]. However, several viruses of the H7 subtype have been shown to replicate efficiently in the mouse model without any prior adaptation [42–44,47]. Such a strain able to infect both birds and mammals is therefore ideal to study and better understand the host specificities related to PB1-F2.

The primary objective of our study was to investigate the virulence of PB1-F2 in relation to strain and host specificities. Our data confirm that the functions of PB1-F2 are different between avian and mammalian hosts. It had already been shown that PB1-F2 expression in chickens tends to reduce the virulence of strains [29,30]. In our work, we observed no difference between WT and ΔF2 H7N1 infected chickens in terms of mortality, viral loads, host response, and tissue tropism. While no effect could be seen in the avian host, infection of mice revealed a clear implication of PB1-F2 from the H7N1 virus in the host inflammatory response as previously published with H1N1 and H5N1 strains [18–20,48,49]. These differences were not observed in vitro since infection of human cells with either WT or ΔF2 H7N1 viruses induced responses of comparable magnitudes. The virulence property of the H7N1 PB1-F2 therefore seems to be effective only within a complex tissue such as the lungs.

It is interesting to note that a viral protein that does not develop any apparent signs of pathology in its original host can behave as a virulence factor after crossing the species barrier. It can be assumed that its functionality could not be exercised in its new host due to the absence of an essential element, such as host interactors. PB1-F2 has been described for its ability to polymerize into fibrillar structures in the vicinity of membranes [50,51], thereby acquiring membrane-lytic activities [52]. As a result, the absence of natural interactors could promote the accumulation of these fibrillar structures in infected cells, inducing membrane disruptions that in turn promote inflammation. However, the situation in the H3N2 context is obviously more complex since the expression of avian PB1-F2 (infection with the chimeric H3N2w/PB1 H7 virus) does not confer the virulence function carried by the avian PB1-F2. These results may appear contradictory at first sight, but could actually reflect a functional dependence of PB1-F2 on a viral component that is present only in its original virus. Alternatively, PB1-F2 could also exert an effect dependent upon a host factor specifically triggered by a virus. A consequence of these findings is that the virulence determinisms of PB1-F2 are probably not only carried by its protein sequence but seem to rely on other factors depending on the viral strain and the nature of the host. As a result, reassortment leading to the integration of an avian segment #2 into an H3N2 background may not be sufficient to confer the whole virulence potential associated with PB1-F2 and the #2 segment.

Recently, HAX-1 was described by two different groups to interact with PB1-F2 [53,54]. HAX-1 is an enigmatic protein which has been described to bind to the nuclear localization signal domain of PA, thereby preventing its nuclear localization and reducing the replication of the virus [55]. While some PA of mammal-adapted viruses are resistant to the restriction effect of HAX-1, zoonotic viruses of avian origin remain sensitive to it [54]. However, through competitive interaction with HAX-1, PB1-F2 of zoonotic viruses can counteract HAX-1’s restriction effect exerted on PA [54]. This specificity therefore contributes to the species barrier and it would be interesting to verify whether the PB1-F2s of the H7N1 and H3N2 viruses studied in our work are capable of binding to HAX-1. Such a mechanism is unlikely to explain our results, given the similar replication abilities of the two viruses. However, it cannot be completely excluded, as an interaction of PB1-F2 with HAX-1 could mobilize other mechanisms associated with HAX-1 such as its recently described links with the NF-kB transduction pathway [56]. We are currently characterizing the interactomes of these two PB1-F2 in order to better understand their respective mechanisms of action. We believe that the key to the puzzles associated with PB1-F2 lies in its own interactome within the infected cell. Its ability to self-assemble in a hydrophobic environment and to destabilize membrane structures also seems to us an essential element in the biology of PB1-F2, since this event could potentially be favored by the lack of its adequate interactors.

## Supplementary Material

Supplemental Material

## Data Availability

The data that support the findings of this study are openly available in figshare at http://doi.org/10.6084/m9.figshare.13857047.
