# Supplementary figures and images for: Study of the host specificity of PB1-F2-associated virulence
Source: Virulence. 2021 Jun 14;12(1):1647–60. doi: 10.1080/21505594.2021.1933848 (PMC8205076; doi:10.1080/21505594.2021.1933848)

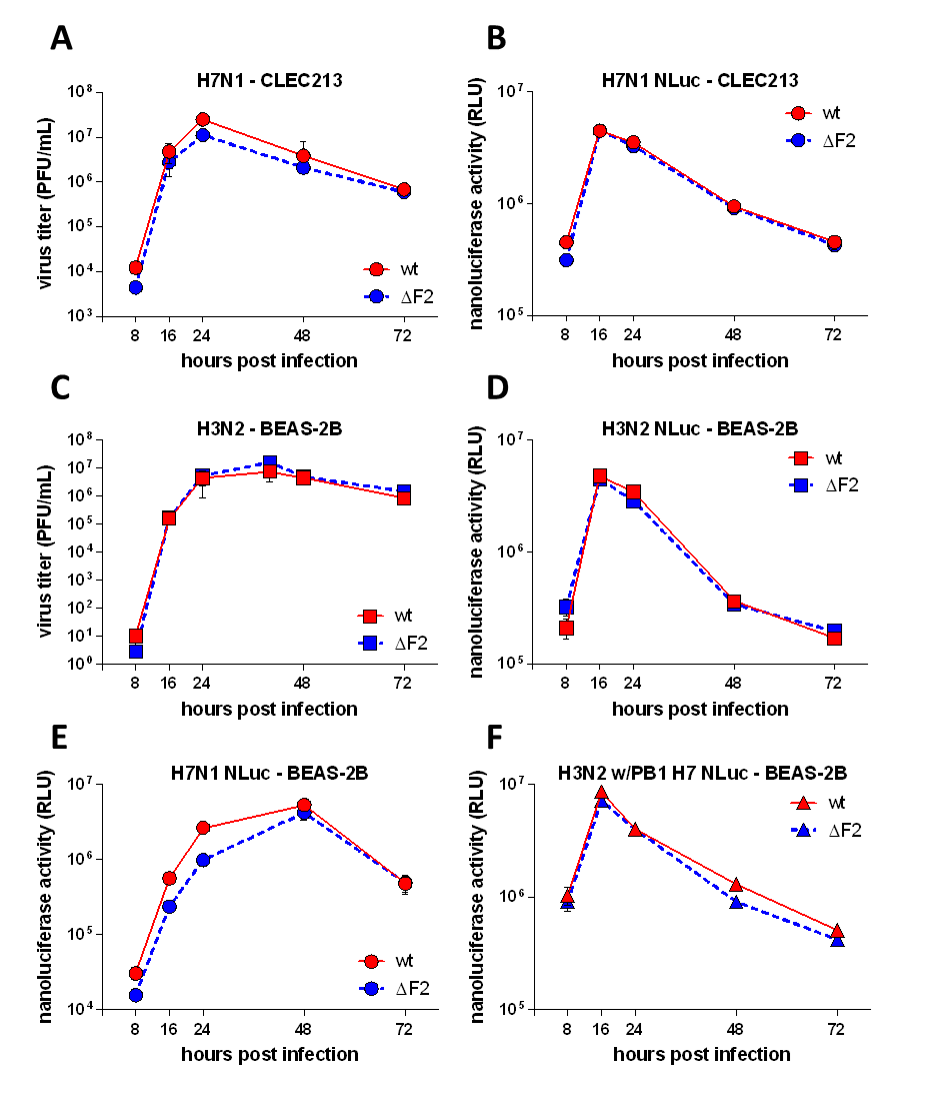

Supplement: Supplemental Material [file KVIR_A_1933848_SM5072.png]
